# 2-(2-Meth­oxy-5-methyl­phen­yl)-2*H*-benzotriazole

**DOI:** 10.1107/S1600536810031363

**Published:** 2010-08-11

**Authors:** Ming-Jen Chen, Chen-Yu Li, Chen-Yen Tsai, Bao-Tsan Ko

**Affiliations:** a Department of Applied Cosmetology and Graduate Institute of Cosmetic Science, Hungkuang University, Taichung Hsien 433, Taiwan; b Department of Chemistry, Chung Yuan Christian University, Chung-Li 320, Taiwan

## Abstract

In the title mol­ecule, C_14_H_13_N_3_O, the dihedral angle between the mean planes of the benzotriazole ring system and the benzene ring is 57.8 (2)°.

## Related literature

For related structures, see: Li *et al.* (2009 ▶, 2010 ▶); Liu *et al.* (2009 ▶). 
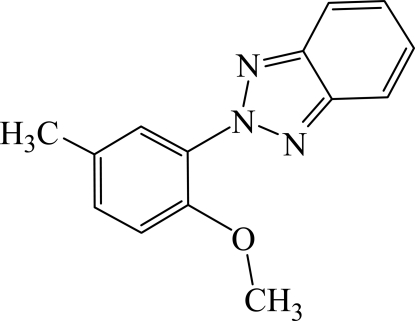


## Experimental

### 

#### Crystal data

C_14_H_13_N_3_O
*M* *_r_* = 239.27
Monoclinic, 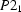

*a* = 7.1604 (2) Å
*b* = 8.2560 (2) Å
*c* = 11.0342 (3) Å
β = 103.450 (1)°
*V* = 634.41 (3) Å^3^
*Z* = 2
Mo *K*α radiation
μ = 0.08 mm^−1^
*T* = 296 K
0.48 × 0.32 × 0.17 mm

#### Data collection

Bruker APEXII CCD diffractometer
Absorption correction: multi-scan (*SADABS*; Bruker, 2008 ▶) *T*_min_ = 0.962, *T*_max_ = 0.986
6057 measured reflections
1674 independent reflections
1401 reflections with *I* > 2σ(*I*)
*R*_int_ = 0.021

#### Refinement

*R*[*F* ^2^ > 2σ(*F* ^2^)] = 0.046
*wR*(*F* ^2^) = 0.136
*S* = 1.05
1674 reflections
164 parameters
1 restraint
H-atom parameters constrained
Δρ_max_ = 0.17 e Å^−3^
Δρ_min_ = −0.15 e Å^−3^

### 

Data collection: *APEX2* (Bruker, 2008 ▶); cell refinement: *SAINT* (Bruker, 2008 ▶); data reduction: *SAINT*; program(s) used to solve structure: *SHELXS97* (Sheldrick, 2008 ▶); program(s) used to refine structure: *SHELXL97* (Sheldrick, 2008 ▶); molecular graphics: *SHELXTL* (Sheldrick, 2008 ▶); software used to prepare material for publication: *SHELXTL*.

## Supplementary Material

Crystal structure: contains datablocks I, global. DOI: 10.1107/S1600536810031363/lh5100sup1.cif

Structure factors: contains datablocks I. DOI: 10.1107/S1600536810031363/lh5100Isup2.hkl

Additional supplementary materials:  crystallographic information; 3D view; checkCIF report

## Figures and Tables

**Fig. 1.**
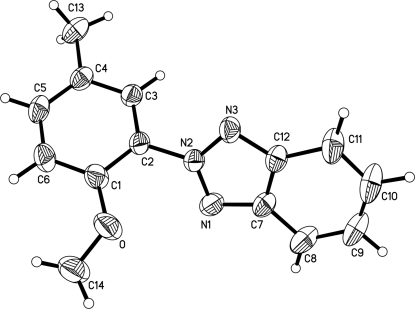
The molecular structure of I with the atom numbering scheme. Displacement ellipsoids are drawn at the 30% probability level.

**Fig. 2.**
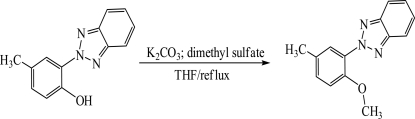
The synthetic procedure of I.

## supplementary crystallographic information

### Comment

In terms of coordination chemistry, the benzotriazole-phenolate (BTP) group can provide the *N, O*-bidentate chelation to stabilize transition metal or main group metal complexes. Therefore, our group is focused on the design and synthesis of the functionalized benzotriazole-phenolate ligand derived from 4-methyl-2-(2*H*-benzotriazol-2-yl)phenol (*^Me^*BTP-H). For instance, our group has successfully synthesized and structural characterized the amino-phenolate ligand *via* a Mannich condensation derived from *^Me^*BTP-H (Li *et al.*, 2009). Most recently, we also reported the synthesis and crystal structure of the salicylaldehyde group substituted benzotriazole derivative (Li *et al.*, 2010). In order to develop more useful ligands originated from BTP derivatives, we report herein the synthesis and crystal structure of the title compound, (I), a potential ligand for the preparation of orthometallated Ir^III^ or Pd^II^ complexes.

The molecular structure of (I) is  shown in Fig. 1. The dihedral angle between the mean planes of the benzotriazole unit and the benzene ring of the 2-methoxy-5-methylphenyl group is 57.8 (2)°, which is larger than that found in the crystal structure of 2-(2*H*-Benzotriazol-2-yl)-4-methylphenyl diphenylphosphinate (Liu *et al.*, 2009).

### Experimental

The title compound (I) was synthesized by the procedure shown in Fig. 2. A mixture of 4-methyl-2-(2*H*-benzotriazol-2-yl)phenol (2.48 g, 10.0 mmol) and potassium carbonate (1.40 g, 10.0 mmol) in THF (30 ml) was stirred at room temperature for 0.5 h. Dimethyl sulfate (1.90 g, 15.0 mmol) was then added and the resulting mixture was refluxed for another 24 h. The mixture was filtered and the filtrate was dried *in vacuo* giving white powder. The white powder was redissolved in hexane and cooled to 253 K to give white crystalline solids. Colourless crystals were obtained from the saturated Et_2_O solution overnight. ^1^H NMR (CDCl_3_, p.p.m.): δ 7.01–7.97 (7*H*, m, Ph*H*), 3.84 (3*H*, s, OC*H*_3_), 2.36 (3*H*, s, C*H*_3_).

### Refinement

In the absence of significant anomalous dispersion effects the Friedel pairs were merged. The H atoms were placed in idealized positions and constrained to ride on their parent atoms, with C–H = 0.93 Å with *U_iso_*(H) = 1.2 *U*_eq_(C) for phenyl hydrogen; 0.96 Å with *U*_iso_(H) = 1.5 *U*_eq_(C) for the CH_3_ groups.

### Crystal data

**Table:**

C_14_H_13_N_3_O	*F*(000) = 252
*M**_r_* = 239.27	*D*_x_ = 1.253 Mg m^−^^3^
Monoclinic, *P*2_1_	Mo *K*α radiation, λ = 0.71073 Å
Hall symbol: P 2yb	Cell parameters from 3727 reflections
*a* = 7.1604 (2) Å	θ = 2.9–28.2°
*b* = 8.2560 (2) Å	µ = 0.08 mm^−^^1^
*c* = 11.0342 (3) Å	*T* = 296 K
β = 103.450 (1)°	Columnar, colourless
*V* = 634.41 (3) Å^3^	0.48 × 0.32 × 0.17 mm
*Z* = 2	

### Data collection

**Table:**

Bruker APEXII CCD diffractometer	1674 independent reflections
Radiation source: fine-focus sealed tube	1401 reflections with *I* > 2σ(*I*)
graphite	*R*_int_ = 0.021
Detector resolution: 8.3333 pixels mm^-1^	θ_max_ = 28.3°, θ_min_ = 1.9°
φ and ω scans	*h* = −8→9
Absorption correction: multi-scan (*SADABS*; Bruker, 2008)	*k* = −11→11
*T*_min_ = 0.962, *T*_max_ = 0.986	*l* = −14→14
6057 measured reflections	

### Refinement

**Table:**

Refinement on *F*^2^	Secondary atom site location: difference Fourier map
Least-squares matrix: full	Hydrogen site location: inferred from neighbouring sites
*R*[*F*^2^ > 2σ(*F*^2^)] = 0.046	H-atom parameters constrained
*wR*(*F*^2^) = 0.136	*w* = 1/[σ^2^(*F*_o_^2^) + (0.0703*P*)^2^ + 0.098*P*] where *P* = (*F*_o_^2^ + 2*F*_c_^2^)/3
*S* = 1.05	(Δ/σ)_max_ < 0.001
1674 reflections	Δρ_max_ = 0.17 e Å^−^^3^
164 parameters	Δρ_min_ = −0.15 e Å^−^^3^
1 restraint	Extinction correction: *SHELXL97* (Sheldrick, 2008), Fc^*^=kFc[1+0.001xFc^2^λ^3^/sin(2θ)]^-1/4^
Primary atom site location: structure-invariant direct methods	Extinction coefficient: 0.067 (13)

### Special details

**Table:**

Geometry. All e.s.d.'s (except the e.s.d. in the dihedral angle between two l.s. planes) are estimated using the full covariance matrix. The cell e.s.d.'s are taken into account individually in the estimation of e.s.d.'s in distances, angles and torsion angles; correlations between e.s.d.'s in cell parameters are only used when they are defined by crystal symmetry. An approximate (isotropic) treatment of cell e.s.d.'s is used for estimating e.s.d.'s involving l.s. planes.
Refinement. Refinement of *F*^2^ against ALL reflections. The weighted *R*-factor *wR* and goodness of fit *S* are based on *F*^2^, conventional *R*-factors *R* are based on *F*, with *F* set to zero for negative *F*^2^. The threshold expression of *F*^2^ > σ(*F*^2^) is used only for calculating *R*-factors(gt) *etc*. and is not relevant to the choice of reflections for refinement. *R*-factors based on *F*^2^ are statistically about twice as large as those based on *F*, and *R*- factors based on ALL data will be even larger.

### Fractional atomic coordinates and isotropic or equivalent isotropic displacement parameters (Å^2^)

**Table:**

	*x*	*y*	*z*	*U*_iso_*/*U*_eq_	
O	0.4845 (3)	0.6862 (5)	0.1130 (2)	0.1062 (12)	
N1	0.3698 (3)	0.9600 (4)	0.2453 (2)	0.0707 (7)	
N2	0.2199 (3)	0.8702 (3)	0.19219 (18)	0.0547 (5)	
N3	0.0939 (3)	0.8349 (3)	0.25880 (19)	0.0610 (6)	
C1	0.3261 (4)	0.7274 (4)	0.0259 (2)	0.0661 (8)	
C2	0.1880 (4)	0.8191 (4)	0.0643 (2)	0.0551 (6)	
C3	0.0210 (4)	0.8665 (4)	−0.0174 (2)	0.0563 (6)	
H3B	−0.0689	0.9279	0.0113	0.068*	
C4	−0.0144 (4)	0.8234 (4)	−0.1425 (2)	0.0602 (7)	
C5	0.1215 (5)	0.7297 (4)	−0.1801 (2)	0.0668 (8)	
H5A	0.0995	0.6976	−0.2630	0.080*	
C6	0.2882 (5)	0.6826 (5)	−0.0987 (3)	0.0720 (8)	
H6A	0.3768	0.6198	−0.1274	0.086*	
C7	0.3395 (4)	0.9863 (4)	0.3605 (2)	0.0638 (7)	
C8	0.4473 (6)	1.0761 (6)	0.4615 (3)	0.0895 (11)	
H8A	0.5613	1.1274	0.4574	0.107*	
C9	0.3761 (6)	1.0840 (5)	0.5651 (3)	0.0949 (13)	
H9A	0.4439	1.1419	0.6336	0.114*	
C10	0.2044 (8)	1.0082 (6)	0.5726 (3)	0.1011 (13)	
H10A	0.1620	1.0180	0.6458	0.121*	
C11	0.0972 (7)	0.9208 (6)	0.4769 (3)	0.0900 (11)	
H11A	−0.0172	0.8715	0.4825	0.108*	
C12	0.1694 (4)	0.9090 (4)	0.3677 (2)	0.0609 (7)	
C13	−0.1948 (5)	0.8824 (5)	−0.2321 (3)	0.0846 (10)	
H13A	−0.2701	0.9448	−0.1875	0.127*	
H13B	−0.2683	0.7911	−0.2707	0.127*	
H13C	−0.1605	0.9487	−0.2951	0.127*	
C14	0.6487 (5)	0.6546 (7)	0.0835 (4)	0.0985 (14)	
H14A	0.7439	0.6277	0.1575	0.148*	
H14B	0.6896	0.7481	0.0448	0.148*	
H14C	0.6321	0.5649	0.0266	0.148*	

### Atomic displacement parameters (Å^2^)

**Table:**

	*U*^11^	*U*^22^	*U*^33^	*U*^12^	*U*^13^	*U*^23^
O	0.0727 (13)	0.190 (4)	0.0590 (11)	0.0505 (19)	0.0224 (10)	0.0159 (18)
N1	0.0598 (13)	0.096 (2)	0.0548 (12)	−0.0144 (13)	0.0095 (10)	−0.0005 (13)
N2	0.0544 (11)	0.0688 (14)	0.0417 (9)	−0.0028 (10)	0.0126 (8)	0.0001 (9)
N3	0.0703 (13)	0.0706 (14)	0.0464 (10)	−0.0090 (12)	0.0222 (9)	−0.0029 (10)
C1	0.0627 (15)	0.089 (2)	0.0502 (13)	0.0096 (16)	0.0203 (11)	0.0099 (14)
C2	0.0568 (13)	0.0702 (16)	0.0403 (10)	−0.0049 (12)	0.0154 (9)	−0.0003 (11)
C3	0.0576 (13)	0.0615 (15)	0.0495 (12)	−0.0047 (12)	0.0122 (10)	−0.0021 (11)
C4	0.0687 (15)	0.0617 (15)	0.0475 (11)	−0.0131 (13)	0.0080 (10)	0.0025 (12)
C5	0.089 (2)	0.0702 (17)	0.0435 (12)	−0.0138 (16)	0.0213 (12)	−0.0059 (12)
C6	0.085 (2)	0.084 (2)	0.0560 (14)	0.0083 (17)	0.0333 (14)	−0.0004 (15)
C7	0.0725 (16)	0.0697 (17)	0.0443 (12)	−0.0040 (14)	0.0037 (11)	0.0046 (12)
C8	0.094 (2)	0.103 (3)	0.0595 (17)	−0.018 (2)	−0.0068 (15)	−0.0009 (19)
C9	0.134 (3)	0.094 (3)	0.0441 (14)	−0.012 (3)	−0.0043 (17)	−0.0044 (16)
C10	0.166 (4)	0.094 (3)	0.0461 (15)	−0.013 (3)	0.0296 (19)	−0.0035 (16)
C11	0.129 (3)	0.094 (2)	0.0549 (16)	−0.021 (2)	0.0374 (18)	−0.0089 (17)
C12	0.0783 (17)	0.0611 (15)	0.0427 (11)	−0.0037 (13)	0.0132 (11)	0.0039 (11)
C13	0.090 (2)	0.095 (3)	0.0565 (15)	−0.009 (2)	−0.0091 (14)	−0.0017 (17)
C14	0.0672 (19)	0.143 (4)	0.091 (2)	0.017 (2)	0.0301 (17)	0.015 (3)

### Geometric parameters (Å, °)

**Table:**

O—C14	1.317 (4)	C7—C12	1.393 (4)
O—C1	1.348 (3)	C7—C8	1.410 (4)
N1—N2	1.324 (3)	C8—C9	1.357 (5)
N1—C7	1.355 (4)	C8—H8A	0.9300
N2—N3	1.322 (3)	C9—C10	1.399 (6)
N2—C2	1.439 (3)	C9—H9A	0.9300
N3—C12	1.345 (3)	C10—C11	1.360 (6)
C1—C2	1.387 (4)	C10—H10A	0.9300
C1—C6	1.389 (4)	C11—C12	1.421 (4)
C2—C3	1.377 (4)	C11—H11A	0.9300
C3—C4	1.391 (4)	C13—H13A	0.9600
C3—H3B	0.9300	C13—H13B	0.9600
C4—C5	1.380 (4)	C13—H13C	0.9600
C4—C13	1.513 (4)	C14—H14A	0.9600
C5—C6	1.372 (4)	C14—H14B	0.9600
C5—H5A	0.9300	C14—H14C	0.9600
C6—H6A	0.9300		
			
C14—O—C1	121.7 (2)	C9—C8—C7	116.6 (4)
N2—N1—C7	102.4 (2)	C9—C8—H8A	121.7
N3—N2—N1	117.7 (2)	C7—C8—H8A	121.7
N3—N2—C2	120.5 (2)	C8—C9—C10	122.5 (3)
N1—N2—C2	121.7 (2)	C8—C9—H9A	118.8
N2—N3—C12	102.2 (2)	C10—C9—H9A	118.8
O—C1—C2	117.5 (2)	C11—C10—C9	122.4 (4)
O—C1—C6	125.2 (3)	C11—C10—H10A	118.8
C2—C1—C6	117.3 (3)	C9—C10—H10A	118.8
C3—C2—C1	121.9 (2)	C10—C11—C12	116.4 (4)
C3—C2—N2	118.3 (2)	C10—C11—H11A	121.8
C1—C2—N2	119.8 (2)	C12—C11—H11A	121.8
C2—C3—C4	120.5 (3)	N3—C12—C7	109.4 (2)
C2—C3—H3B	119.8	N3—C12—C11	129.7 (3)
C4—C3—H3B	119.8	C7—C12—C11	120.8 (3)
C5—C4—C3	117.6 (3)	C4—C13—H13A	109.5
C5—C4—C13	122.6 (3)	C4—C13—H13B	109.5
C3—C4—C13	119.8 (3)	H13A—C13—H13B	109.5
C6—C5—C4	121.9 (2)	C4—C13—H13C	109.5
C6—C5—H5A	119.0	H13A—C13—H13C	109.5
C4—C5—H5A	119.0	H13B—C13—H13C	109.5
C5—C6—C1	120.8 (3)	O—C14—H14A	109.5
C5—C6—H6A	119.6	O—C14—H14B	109.5
C1—C6—H6A	119.6	H14A—C14—H14B	109.5
N1—C7—C12	108.2 (2)	O—C14—H14C	109.5
N1—C7—C8	130.4 (3)	H14A—C14—H14C	109.5
C12—C7—C8	121.4 (3)	H14B—C14—H14C	109.5
			
C7—N1—N2—N3	0.3 (3)	C13—C4—C5—C6	177.3 (3)
C7—N1—N2—C2	177.1 (3)	C4—C5—C6—C1	0.3 (5)
N1—N2—N3—C12	−0.2 (3)	O—C1—C6—C5	179.4 (3)
C2—N2—N3—C12	−177.0 (2)	C2—C1—C6—C5	0.9 (5)
C14—O—C1—C2	−154.6 (4)	N2—N1—C7—C12	−0.3 (3)
C14—O—C1—C6	26.9 (7)	N2—N1—C7—C8	−178.9 (4)
O—C1—C2—C3	−179.7 (3)	N1—C7—C8—C9	178.0 (4)
C6—C1—C2—C3	−1.0 (5)	C12—C7—C8—C9	−0.4 (5)
O—C1—C2—N2	1.8 (4)	C7—C8—C9—C10	−0.3 (6)
C6—C1—C2—N2	−179.5 (3)	C8—C9—C10—C11	0.3 (7)
N3—N2—C2—C3	56.9 (4)	C9—C10—C11—C12	0.4 (7)
N1—N2—C2—C3	−119.8 (3)	N2—N3—C12—C7	0.0 (3)
N3—N2—C2—C1	−124.6 (3)	N2—N3—C12—C11	177.5 (4)
N1—N2—C2—C1	58.8 (4)	N1—C7—C12—N3	0.2 (4)
C1—C2—C3—C4	−0.1 (4)	C8—C7—C12—N3	178.9 (3)
N2—C2—C3—C4	178.5 (3)	N1—C7—C12—C11	−177.6 (3)
C2—C3—C4—C5	1.2 (4)	C8—C7—C12—C11	1.1 (5)
C2—C3—C4—C13	−177.5 (3)	C10—C11—C12—N3	−178.4 (4)
C3—C4—C5—C6	−1.3 (5)	C10—C11—C12—C7	−1.1 (6)
